# Safety and cost-effectiveness of single-use endolaser probe reprocessing in vitreoretinal surgery

**DOI:** 10.1186/s40942-021-00292-x

**Published:** 2021-03-17

**Authors:** Leandro Cabral Zacharias, Lívia da Silva Conci, Bianca Partezani Megnis, Janaina Guerra Falabretti, Taurino dos Santos Rodrigues Neto, Epitácio Dias da Silva Neto, Rony Carlos Preti, Leonardo Proveti Cunha, Mário Luiz Ribeiro Monteiro

**Affiliations:** Department of Ophthalmology, University of Sao, Paulo255 Dr. Enéas de Carvalho Aguiar Avenue, Sao Paulo, SP 05403000 Brazil

**Keywords:** Vitrectomy, Laser Coagulation, Sterilization, Cost–Benefit Analysis

## Abstract

**Background:**

Endolaser probes have been designed and sold for single-use only. However, in Brazil, they are not included in the list of single-use medical products that are prohibited from being reprocessed and could potentially be reused if safety requirements are accomplished. Therefore, this study aimed to determine and compare the quality, safety and costs of reprocessed versus original single-use endolaser probes of a specific brand and model.

**Methods:**

The study, conducted at a university hospital in Sao Paulo, Brazil, was divided in two phases. The first one tested the feasibility, sterility and physical integrity of ten reprocessed laser probes. In the second phase, all vitrectomy procedures using endolaser probes (reprocessed and original ones) from August 2017 to October 2019 were evaluated. The operated cases were followed for any signs of infection and number of defective probes for each group were counted. The cost of acquiring a new probe and for all reprocessing stages were evaluated and quantified in US dollars($).

**Results:**

Microbiologic, residual ethilen oxide and microscopic evaluation of integrity of reprocessed laser probes were all within acceptable range. The second phase of this study included 590 endolaser probes, of which 375 were original and 215 were reprocessed. Functionality rates differed significantly between groups. Among the original probes, 373 (99.47%) were functioning and 2 (0.53%) were non-functioning. Among the reprocessed ones, 201 (93.5%) were functioning and 14 (6.5%) were non-functioning (p < .001). The average cost of one reprocessing was $3.00, and the average cost of an original probe was $150.00. Considering the loss rates, potential savings were $147.60 for each once-reprocessed probe. The frequency of infectious endophthalmitis was null in both groups.

**Conclusions:**

Our study showed that a single cycle endolaser probe reprocessing was safe and efficient, not associated with increase in endophthalmitis rate and proved to be significantly cost-effective, even considering a greater malfunction rate when compared to the original devices.

## Background

First reported in 1981 [1], the introduction of endophotocoagulation was a significant advance in vitreoretinal surgery and, since then, many laser probe models have been developed for this use [2]. Endolaser probes are a useful tool for the induction of chorioretinal adhesions and to ablate ischemic retina. Therefore, this instrument plays an important role in the surgical treatment of common diseases, such as rhegmatogenous retinal detachment, retinal tears and proliferative diabetic retinopathy [3].

Despite having no lumen, endolaser probes have been designed and sold for single-use only. In Brazil, the National Health Surveillance Agency (ANVISA) has been responsible for the regulation of medical products reprocessing [4]. According to its resolution number 2605, published on August 11th, 2006, endolaser probes are not included in the list of single-use medical products that are prohibited from being reprocessed and therefore could potentially be reprocessed [5]. Moreover, the fact that probe design has no internal lumen theoretically allows proper cleaning and sterilization. In general, the cost to reprocess each device is significantly lower than the cost of purchasing an original one [6]. However, there is no available information regarding the sterilization protocol, safety or percentage of malfunction related to endolaser probe reprocessing process. Adding to that, many manufacturers add a single use label on the packing, that inhibits any reprocessing initiative.

The present study aimed to establish safety, functionality and cost-effectiveness of reprocessing laser probes of a specific brand and model. Therefore, a two-phase study was conducted; the first phase focused on microbiologic and integrity tests; the second phase prospectively evaluated and compared patients submitted to pars plana vitrectomy with either original or reprocessed endolaser probes.

## Methods

This study was conducted at the University of Sao Paulo Medical School Clinics Hospital (HCFMUSP), located in Sao Paulo-SP, Brazil, and it was approved by the Institutional Review Board, under registration number 31251520.1.0000.0068. All vitrectomy procedures from August 2017 to October 2019 in which surgeon used endolaser probes were included in the study.

**Fig. 1:**
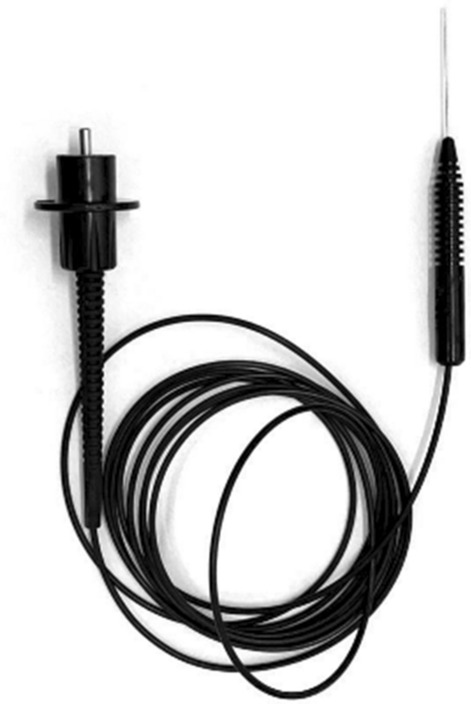
23 Ga Straight Laser Probe (Alcon Laboratories, Inc., FortWorth, TX)

At HCFMUSP, the endolaser probes used (23 Gauge Straight Laser Probe) are compatible with the Constellation vitrectomy system and belong to the Purepoint 532 nm thin disc laser system (Alcon Laboratories, Inc., FortWorth, TX). They are terminated at one side with a connector that attaches the fiber end to the laser source. At the other side, they are terminated with a handpiece and a 23-gauge stainless steel tubing (without lumen) that holds the straight tip of the fiber. A flexible plastic jacket covers and protects the length of the fiber (Fig. 1).

As these probes are marketed for single use and there is no formal reprocessing guidance, a protocol for endolaser probes reprocessing was implemented after microbiological, residual, and physical integrity tests. In the first phase of the study, ten endolaser probes initially used in vitreoretinal procedures that would be discarded were collected and evaluated for bacterial, yeast, and hyphae count before and after a cleaning process. In addition, they were submitted to a sterilization cycle with 90/10 percent ethylene oxide mixture and sterility was tested. The presence and amount of pyrogen was assessed with a bacterial endotoxin test and measured in units of endotoxin per milliliter (UE/mL). As for the physical–chemical analyzes, the residual ethylene oxide (ETO), ethylene chlorohydrin (ETC), and ethylene glycol (ETG) were quantified by gas chromatography in parts per million (PPM). Integrity assessment was performed using an electron microscope with an increase of up to 200 times.

The second phase of the study was designed in conjunction with the Hospital Infection Control Committee of our institution. According to the sterilization protocol accorded at the institution, each probe could be resterilized only once. Hence, already reprocessed probes had to be discarded after their intraoperative use.

After each surgery, information such as the use of sterilized or new probes, as well as any malfunction of the probe, was collected. Laser malfunction was defined as the non-visualization of the burns during the intraoperative use that required the use of another probe. The sheet with the information above was filled by the surgeon right after the procedure, and it had the patient identification on it in order to track for any adverse events. All operated cases were carefully followed for any signs of infection for the post-operative period of one year, and any suspicious or confirmed event would have to be reported immediately to the Infection Control Committee of the Hospital.

The number of defective probes for each group (reprocessed versus original probes) were counted and statistically analyzed. The loss rates were calculated by the ratio between the number of non-functioning probes and the total number of probes used in each group. Moreover, the hospital costs of acquiring a new endolaser probe and the cost of all stages of probe reprocessing were evaluated and compared for the whole period. All costs were presented in US dollars ($).

Data analysis included chi-square test for categorical variables. Level of significance was set to p < 0.05. All the analyses were performed by the Statistical Package for Social Sciences (SPSS 20.0; New York, USA) software.

## Results

The results of the first phase of the study are presented in the Table 1. A significant decline in the microorganisms count (bacteria, hyphae and yeasts) was observed after cleaning the probes. In addition, in all ten samples, after the sterilization cycle, the sterility tests were negative for any microbial growth. As for the presence of endotoxins, the amount of pyrogen detected was minimal (< 0.5 EU/mL), and ETO, ETC and ETG residues were within the recommended safe limit (< 25PPM, < 25PPM, and < 250PPM, respectively). Electron microscopy probe integrity was also satisfactory for all samples, with no irregularities in structure, cracks or bubbles detected.

**Table 1:** Microbiological, Residual and Physical Integrity Tests Results

Endolaser probes samples	Bacterial count (CFU)		Yeast count (CFU)		Hyphae count (CFU)		Sterility test	Bacterial endotoxin test	Physical–chemical analyzes			Physical integrity
	Before cleaning	After cleaning	Before cleaning	After cleaning	Before cleaning	After cleaning	Microbial growth	Amount of pyrogen (EU/mL)	ETO residues (PPM)	ETC residues (PPM)	ETG residues (PPM)	Assessment with a microscope (increase of up to 200 times)
Number 1	3	1	1	1	1	1	Ausent	0.050	20.77	2.30	6.49	Without changes
Number 2	15	10	1	1	3	2	Ausent	0.050	14.51	0.50	10.01	Without changes
Number 3	25	2	1	1	1	1	Ausent	0.073	13.49	1.34	3.09	Without changes
Number 4	53	10	1	1	1	1	Ausent	0.053	22.79	2.29	2.52	Without changes
Number 5	36	4	1	1	1	1	Ausent	0.059	10.48	2.06	13.02	Without changes
Number 6	24	3	1	1	1	1	Ausent	0.050	10.77	0.65	0.64	Without changes
Number 7	30	7	1	1	1	1	Ausent	0.050	17.72	0.80	0.75	Without changes
Number 8	18	1	1	1	1	1	Ausent	0.050	19.39	0.85	0.50	Without changes
Number 9	64	23	1	1	1	1	Ausent	0.150	17.62	0.69	0.74	Without changes
Number 10	30	8	1	1	1	1	Ausent	0.250	13.01	2.63	1.75	Without changes

**Fig. 2:**
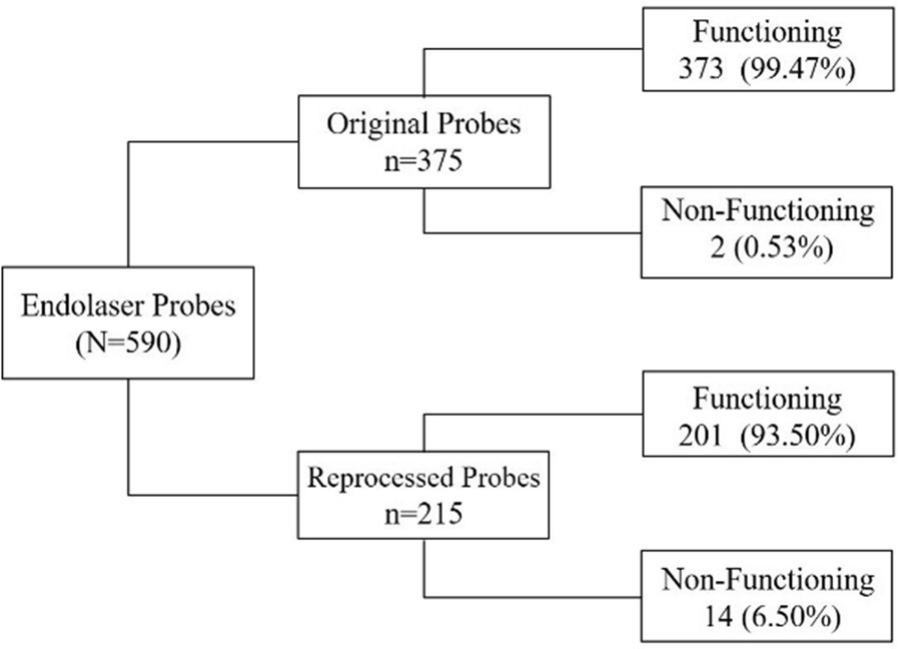
Schematic representation of the evaluated probes

The second phase of this study included 590 endolaser probes used during vitrectomy surgeries performed in our institution between August 2017 and October 2019, of which 375 (63.56%) were original and 215 (36.44%) were reprocessed. Functionality rates differed significantly between the groups (OR = 12.99; p < 0.001). Among the original probes, 373 (99.47%) were functioning and 2 (0.53%) presented malfunction, while among the reprocessed ones, 201 (93.5%) were properly functioning and 14 (6.5%) were not (Fig. 2). The malfunctioning was reported by the surgeon in a real-life scenario during vitrectomy surgery, and no problems related to light emission errors were reported. None of the probes were reported as broken during sterilization process.

The average cost of one reprocessing cycle was $3.00 and the average cost of an original probe was $150.00. Considering the loss rates, potential savings were $147.60 for each once-reprocessed probe.

Regarding to safety, no cases of endophthalmitis were observed in both groups after one year of prospective follow up.

## Discussion

Any device used in healthcare practice carries a certain degree of risk and can cause problems under certain conditions, and therefore, there is no absolute safety regarding medical devices. In laser devices, for instance, there is a risk of thermally-induced retinal damage related to exposure above safety thresholds [7]. However, considering that the laser probe consists basically in an optic fiber to conduct and deliver energy, and the original probes were thoroughly tested regarding safety, any emission issues related only to its reprocessing are unexpected. As far as reprocessed devices are concerning, there are two main potential problems: the risk of infections and the loss of product functionality [8]. Despite those risks, the main justification for this practice is the high cost of healthcare devices, what makes reprocessing a common practice and a necessity at most Brazilian healthcare institutions [9]. Moreover, in many cases the non-functionality of a certain surgical device may jeopardize the surgical outcome. That is not the case of endolaser probes, that can be promptly exchanged without any harm in case they do not work properly.

A recent meta-analysis showed that post-23-gauge vitrectomy endophthalmitis incidence is low (0.03%) [10]. Although infrequent, acute infectious endophthalmitis is one of the most feared postoperative complications, and is related to an unfavorable visual outcome [11]. Nevertheless, in practice, the reuse of ophthalmic materials is often performed without any validation as to the safety of their reprocessing [9]. Furthermore, other types of postoperative inflammatory reaction, such as noninfectious (sterile) endophthalmitis and toxic anterior segment syndrome (TASS) may be attributable to retained toxicity following introduction of certain substances into the eye [12, 13]. Given the risks, we believe that reprocessing of ophthalmic single-use devices should be considered only when there is no increased risk of infection or inflammatory reactions related to the presence of endotoxins, toxic residues of cleaning products, material functionality or integrity. For this reason, in our institution, tests were carried out before the routine implementation of the endolaser probe-reprocessing protocol and all samples showed satisfactory results (Table 1).

In the present study, between August 2017 to October 2019, the overall incidence of presumed endophthalmitis following vitrectomy procedures was null in both groups. Thus, the use of reprocessed endolaser probes has not been associated with an additional risk of endophthalmitis in this cohort and based on our findings and following the methods described, one single reprocessing cycle can be safely recommended.

Another Brazilian study evaluated the microbial growth on single-use reprocessed vitrectomy probes (including aspiration lines with lumen) [9]. Without a standardized cleaning protocol, the results demonstrated microbial growth on 57/979 (5.8%) sample units and then, the authors did not recommend reprocessing of these probes. A possible explanation for the microbial growth rate would be that the tubing line of the vitrectomy probes does not allow proper cleaning. On the other hand, a more recent study conducted in Thailand adopted a standardized protocol for cleaning disposable devices in vitrectomy, which consisted in the use of enzymatic detergent through the lumen of tubing, rinsing with sterile water, and cleaning with an ultrasonic cleaner [14]. In this real-life study, use of recycled single-use instruments did not seem to increase the risk of endophthalmitis. It is noteworthy, however, that this practice would have restrictions in Brazil, due to our regulatory health organ (ANVISA) legislation, which prohibits the reprocessing of disposable devices with lumen. Certainly, there is a considerable structural advantage of the endolaser probes that make its reprocessing effective and reproductible, as since there are no lumens or connectors.

Regarding the cost analysis, the instruments’ functionality is an important factor. In the group of reprocessed probes, there was a higher loss of function rate (6.50%) when compared to the group of original probes (0.53%), OR = 12.99, p < 0.001. We believe this functionality loss is related to the strict cleaning process implemented in the sterilization process, that may damage the optic fibers responsible for laser transmission. Even though, reprocessed probes exhibited great cost-effectiveness, with savings of $147.60 for each reprocessed probe. In the context of public health, these savings can be socially impactful, as they allow investment in other devices and even in an increasing number of procedures [15]. Moreover, the current world pandemic may require a wise allocation of public health care funds and nowadays environmental, social and governance (ESG) issues are hot topics discussed by the society as a hole, and therefore the decrease environmental impact of proper device sterilization should also be considered.

## Conclusions

In summary, our study showed that the one-cycle reprocessing of endolaser probes was safe and efficient, since it has not contributed to an increase in endophthalmitis cases and it was significantly cost-effective, even with greater functionality loss rates.

## Data Availability

All data generated or analyzed during this study are included in this published article.

## Abbreviations

EU/mL: Units of endotoxin per milliliter
ETO: Ethylene oxide
ETC: Ethylene chlorohydrin
ETG: Ethylene glycol
PPM: Parts per million
CFU: Colony-forming unit
